# Peripherally restricted PICK1 inhibitor mPD5 ameliorates pain behaviors in murine inflammatory and neuropathic pain models

**DOI:** 10.1172/jci.insight.170976

**Published:** 2024-09-17

**Authors:** Kathrine Louise Jensen, Nikolaj Riis Christensen, Carolyn Marie Goddard, Sara Elgaard Jager, Gith Noes-Holt, Ida Buur Kanneworff, Alexander Jakobsen, Lucía Jiménez-Fernández, Emily G. Peck, Line Sivertsen, Raquel Comaposada Baro, Grace Anne Houser, Felix Paul Mayer, Marta Diaz-delCastillo, Marie Løth Topp, Chelsea Hopkins, Cecilie Dubgaard Thomsen, Ahmed Barakat Ibrahim Soltan, Frederik Grønbæk Tidemand, Lise Arleth, Anne-Marie Heegaard, Andreas Toft Sørensen, Kenneth Lindegaard Madsen

**Affiliations:** 1 Molecular Neuropharmacology and Genetics Laboratory, Department of Neuroscience,; 2 Center for Biopharmaceuticals, Department of Drug Design and Pharmacology, and; 3 Department of Drug Design and Pharmacology, Faculty of Health and Medical Sciences, University of Copenhagen, Copenhagen, Denmark.; 4 Department of Translational Neuroscience, Wake Forest University School of Medicine, Winston-Salem, North Carolina, USA.; 5 X-ray and Neutron Science, Niels Bohr Institute, Faculty of Science, University of Copenhagen, Copenhagen, Denmark.

**Keywords:** Neuroscience, Pain, Peptides, Pharmacology

## Abstract

Chronic pain is a complex, debilitating, and escalating health problem worldwide, impacting 1 in 5 adults. Current treatment is compromised by dose-limiting side effects, including high abuse liability, loss of ability to function socially and professionally, fatigue, drowsiness, and apathy. PICK1 has emerged as a promising target for the treatment of chronic pain conditions. Here, we developed and characterized a cell-permeable fatty acid–conjugated bivalent peptide inhibitor of PICK1 and assessed its effects on acute and chronic pain. The myristoylated PICK1 inhibitor, myr-NPEG_4_-(HWLKV)_2_ (mPD5), self-assembled into core-shell micelles that provided favorable pharmacodynamic properties and relieved evoked mechanical and thermal hypersensitivity as well as ongoing hypersensitivity and anxiodepressive symptoms in mouse models of neuropathic and inflammatory pain following subcutaneous administration. No overt side effects were associated with mPD5 administration, and it had no effect on acute nociception. Finally, neuropathic pain was relieved far into the chronic phase (18 weeks after spared nerve injury surgery) and while the effect of a single injection ceased after a few hours, repeated administration provided pain relief lasting up to 20 hours after the last injection.

## Abstract 2

mPD5 is a peripherally restricted peptide drug that relieves both ongoing and evoked hypersensitivity in multiple mouse models of pain in female and male mice.

## Graphical abstract

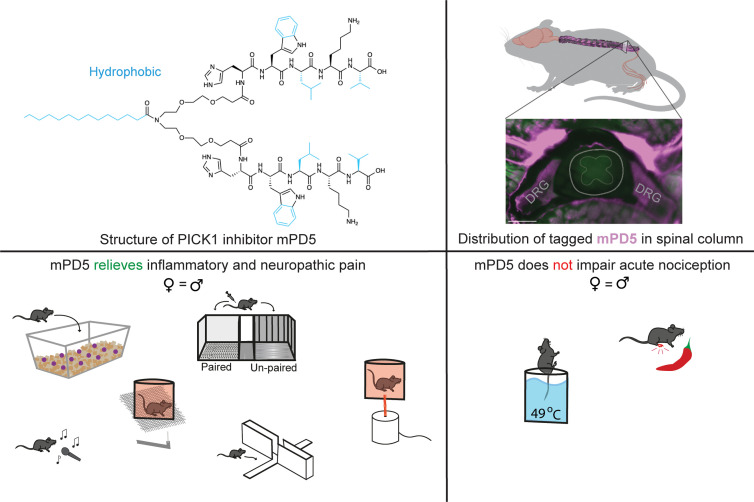

## Introduction

The International Classification of Diseases 11th Revision (ICD-11) defines chronic pain (MG30) as a multifactorial syndrome of pain persisting for more than 3 months with psychological, biological, and social factors contributing to the syndrome (1). Despite complying with recommended treatment guidelines, a large fraction of the 1.5 billion people suffering from chronic pain experience compromised quality of life, with constant pain and impairment of work and social life (2, 3). First-line treatments show very low efficacy of chronic pain relief, with numbers needed to treat ranging from 6 to 10 depending on the aetiology of the pain condition (3–5), while opioid-based treatments entail a significant risk of high opioid use and abuse (6). The lack of efficacy and severe dose-limiting side effects originating from their centrally modulating pain transmission of current treatments highlight an urgent need to develop more effective, non-addictive pain therapeutics.

An emerging strategy for alleviating pain is modulation of receptor trafficking by targeting specific scaffold proteins (7–10). Protein interacting with C kinase 1 (PICK1) is a PDZ domain–containing scaffold protein enriched in the postsynaptic density of neurons, known for its role in central synaptic plasticity (11, 12) and hormone storage and release (13, 14). PICK1 interacts with a host of membrane proteins and kinases via its PDZ domain (15–17), many of which have been implicated in pain signalling (18). Notably, PICK1 regulates subcellular localization and surface expression of its interaction partners, including the GluA2 subunit–containing α-amino-3-hydroxy-5-methyl-4-isoxazolepropionic acid (AMPA) type glutamate receptors (AMPARs) (11) and acid-sensing ion channels (ASICs) (19). PICK1 is, among other places, expressed in areas important for the transmission of painful stimuli, such as the dorsal root ganglia (DRGs) and the dorsal horn (concentrated in lamina I and the inner lamina II) (7, 10, 20, 21). Based on studies in animal models using inhibitory peptides, siRNA, and knockout mice, PICK1 has been shown to be implicated in thermal and mechanical hypersensitivity in neuropathic and inflammatory pain models, suggesting PICK1 as a putative target for pharmaceutical intervention of chronic pain states (7, 8, 20–22).

Developing small molecule inhibitors of PICK1 has proven difficult (17, 23, 24), with some progress in the last decade (25–27). Our recent development of a membrane permeable, bivalent, high-affinity PICK1 inhibitor, TPD5 (8), displaying low nanomolar target affinity, represents a major leap toward a potential PICK1-targeting therapeutic. Intrathecal (i.t.) administration of TPD5 fully alleviated mechanical hypersensitivity in the spared nerve injury (SNI) model of neuropathic pain. However, TPD5 was designed to penetrate the blood-brain barrier and target spinal cord plasticity, raising concerns about central side effects, as is known for current treatments (6, 8, 28–30). Nonetheless, TPD5 concomitantly reduced transmission in the Lissauer’s tract, demonstrating effect on the first-order DRG neurons (8). In addition, we have shown that AAV-mediated expression of similar PICK1 inhibitors, confined to DRGs, is sufficient for full pain relief (31). In recent years, the use of fatty acid modifications on peptides has emerged as a successful way to enhance both plasma stability and cell permeability of pharmaceutical peptides, while also offering a benevolent toxicology profile and low CNS exposure (32–34). In the current study, we describe the development of a myristoylated lipid-conjugated peptide PICK1 inhibitor, myr-NPEG_4_-(HWLKV)_2_ (mPD5), thereby circumventing concerns raised over the safety profile of TAT-conjugated peptides, particularly for drugs intended for repeated administration (35, 36) and (TAT NR2B9c, US patent 8,080,518 B2).

mPD5 demonstrated high stability, solubility, and plasma protein binding, as well as low blood-brain-barrier penetrance, all compatible with further drug development. Functional characterization of mPD5 following subcutaneous (s.c.) administration in mice showed robust relief of mechanical and thermal hypersensitivity in mouse models of both inflammatory and neuropathic pain in female and male mice. Moreover, mPD5 reverted anxiodepressive symptoms and ongoing pain without affecting locomotor activity or putative on-target effects on learning, memory, and male fertility. In contrast with other peripherally acting pain-relieving drugs, mPD5 did not affect acute nociception. Finally, repeated administration of mPD5 gave rise to sustained relief of mechanical hypersensitivity lasting 20 hours after the last administration, advancing mPD5 as a strong lead molecule in the clinic for the treatment of chronic pain conditions.

## Results

### mPD5 oligomerizes into micellar structures.

**Figure 1:**
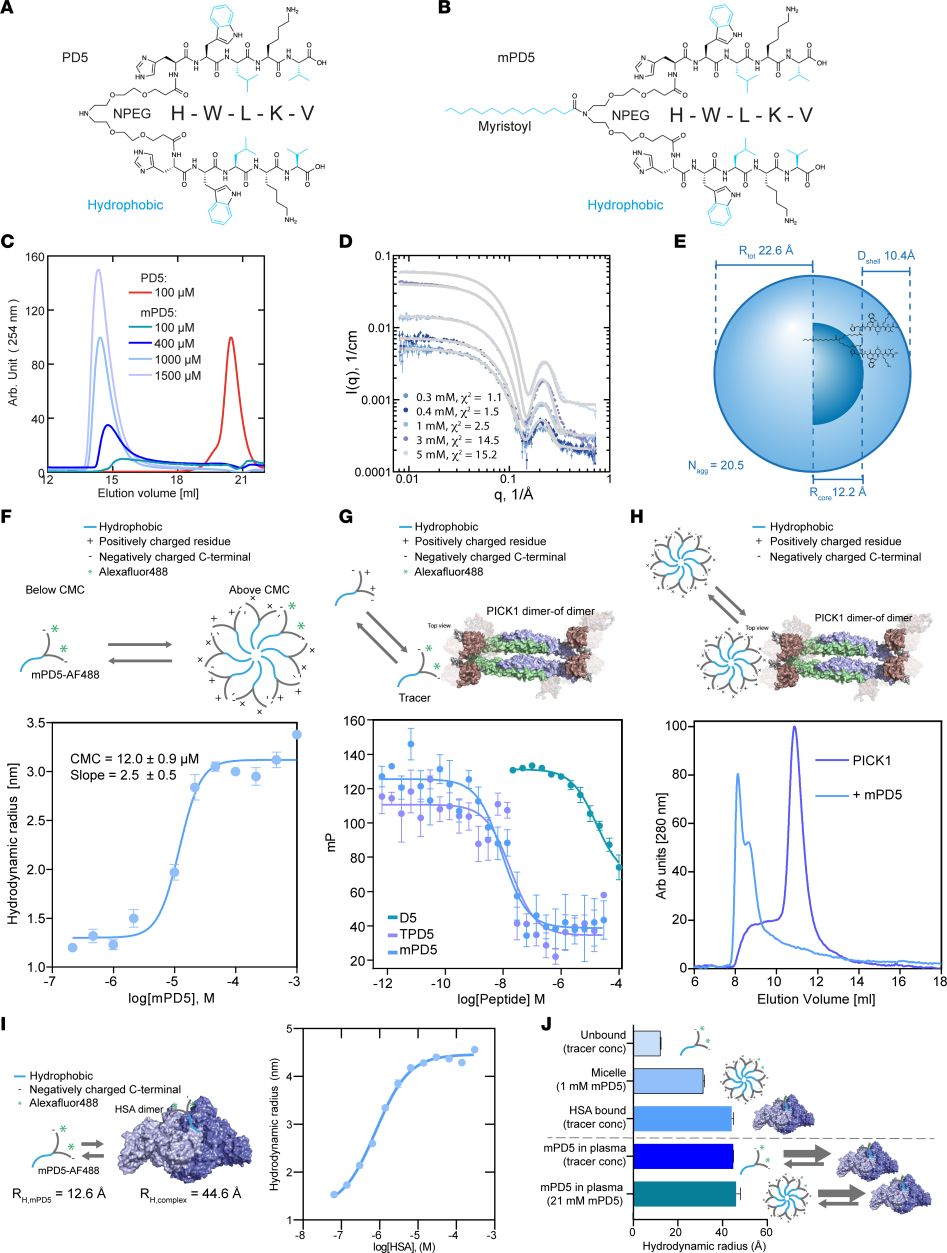
Biophysical characterization of mPD5. Structure of NPEG_4_-(HWLKV)_2_ (PD5) (**A**) and myristoyl-NPEG_4_-(HWLKV)_2_ (mPD5) (**B**) (hydrophobic parts in blue). (**C**) Size exclusion chromatography (SEC) with different concentrations of mPD5 demonstrating self-assembly. (**D**) Small-angle x-ray scattering (SAXS) data (points) of mPD5 at different concentrations. Model fits of the core-shell model (lines) (see Supplemental Table 1 for fitting parameters). l(q) = scattering intensity. (**E**) Molecularly constrained spherical core-shell model fitted to the data in Supplemental Figure 1B reveals 20.5 molecules/micelle: total radius, *R*_total_ = 22.6 Å. (**F**–**H**) Experimental illustration above data. (**F**) Flow-induced dispersion analysis (FIDA) binding isotherm of mPD5 combining optimal coatings for different concentrations (additional information in Supplemental Figure 2); CMC = 12 μM; hydrodynamic radius, *R*_H_ ≈ 30 Å. (**G**) Fluorescence polarization (FP) competition binding curves of mPD5 (blue) (*K*_i,app_ = 3.0 nM, SEM interval [2.3–3.8] nM, *n* = 6), TPD5 (purple) (*K*_i,app_ = 3.9 nM, SEM interval [3.5–4.4] nM, *n* = 3) and D5 (HWLKV) (green) (*K*_i,app_ = 6998 nM, SEM interval [4972–9849] nM, *n* = 3), using 5FAM-PD5 (5 nM vs. TPD5 and mPD5) or 5FAM-D5 (20 nM vs. D5) as tracer. Data were fitted to a competitive binding 1-site fit using GraphPad Prism 8.3. (**H**) SEC elution profile of PICK1 in the absence (purple) or presence (blue) of mPD5, at a PICK1/mPD5 molecular ratio of 4:1. (**I**) Isotherm of mPD5-AF488 binding to human serum albumin (HSA); affinity (*K*_D_) = 787 nM; hydrodynamic radius (*R*_H_) of the complex = 4.46 nm. Experimental illustration is to the left of the data. (**J**) Histogram showing the *R*_H_s ± SEM of mPD5 (*n* = 3).

We have previously developed and characterized the blood-brain-barrier permeable bivalent PICK1 peptide inhibitor, TAT-NPEG_4_-(HWLKV)_2_ (TPD5), showing promise as a therapeutic lead for the treatment of pain (8, 22) and addiction (37). To reduce potential side effects, and to facilitate plasma stability and distribution, we introduced a C_14_ fatty acid (myristic acid, myr) instead of the cell-penetrating TAT sequence on the same scaffold, NPEG_4_-(HWLKV)_2_ (PD5) (Figure 1A), resulting in myr-NPEG_4_-(HWLKV)_2_ (mPD5) (Figure 1B). mPD5 demonstrated excellent shelf stability (Table 1) and was soluble to at least 250 mg/mL (~130 mM) in PBS, as judged from a transparent and monophasic appearance.

We hypothesized that these attractive properties might arise from an oligomeric or core-shell micellar self-assembly, with the hydrophobic lipid forming a central core and the hydrophilic PEG_4_-linked pentamer peptide facing the aqueous solution. Using size exclusion chromatography (SEC) (Figure 1C) we found that PD5, lacking the myristic acid, eluted at approximately 21 mL, whereas mPD5 eluted at approximately 14.2 mL, independent of initial concentration, consistent with self-assembly of mPD5 (Figure 1C). We used small-angle x-ray scattering (SAXS) to investigate putative micelle structure and size. Data (Figure 1D and Supplemental Figure 1; supplemental material available online with this article; https://doi.org/10.1172/jci.insight.170976DS1) were characteristic of core-shell micellar particles exhibiting a significant oscillation at high *q*, as well as an extended flat Guinier region at low *q* values, indicating small micelles without presence of larger aggregates. Further analysis of the low *q* region showed that the forward scattering (*I*_0_) of mPD5 scaled linearly with concentration (Supplemental Figure 1A), suggesting concentration-independent particle size with no significant interparticle interactions in the studied concentration range (0.3–5 mM). The pair-distance distribution, *p*(*r*), showed a radius of gyration (*R*_g_) of approximately 30 Å and a *D*_max_ of approximately 50 Å (Supplemental Figure 1C), with a shape consistent with core-shell particles (38, 39). We fitted the SAXS data using a molecularly constrained model for spherical core-shell micelles composed of an inner core of the fatty acid part of mPD5 and an outer shell of the peptide part of mPD5 (Supplemental Figure 1B and Figure 1E) (full model account in Methods in supplemental information). The fitting suggested that on average, approximately 20 mPD5 molecules make up the assembled micelle, with a hydrophobic core radius (*R*_core_) of 12.2 Å, a hydrophobic shell thickness (*D*_shell_) of 10.4 Å, and hence a total radius (*R*_total_) of 22.6 Å (Figure 1E and Supplemental Table 1). To determine the critical micelle concentration (CMC) of mPD5, we used flow-induced dispersion analysis (FIDA). FIDA suggested a CMC of 12 μM, and a hydrodynamic radius (*R*_H_) of approximately 30 Å (Figure 1F and Supplemental Figure 2).

### mPD5 binds PICK1 with high affinity.

Using fluorescence polarization competition binding, we determined the affinity (*K*_i,app_) of mPD5 for PICK1 to be 3.0 nM (SEM interval [2.3–3.8] nM), which is similar to TPD5 (*K*_i,app_ = 3.9 nM, SEM interval [3.5–4.4] nM) (8), demonstrating an approximately 1000-fold affinity gain compared with D5 (HWLKV) alone (*K*_i,app_ = 6.9 μM, SEM interval [5.0–9.9] μM) and a 30-fold affinity gain compared with PD5 (*K*_i,app_ = 98 nM) (Figure 1G). This demonstrates that both bivalency and the lipid chain contribute to the overall binding strength. Finally, to evaluate the ability of micellar mPD5 to bind PICK1, we incubated recombinant full-length PICK1 (40 μM) with mPD5 in a concentration corresponding to the CMC (10 μM) and observed a distinct shift in the peak elution volume of PICK1 from 10.9 mL in the absence of mPD5 to 2 peaks at 8 mL and 9 mL in the presence of mPD5 (Figure 1H), suggesting the ability of mPD5 to induce higher-order complexes.

### mPD5 binds to human serum albumin in plasma.

Fatty acids are reported to mediate drug binding to serum albumin, thereby enhancing plasma lifetime due to reduced renal clearance and metabolism (32, 33). Since mPD5 is lipidated and self assembles into micelles, we assessed whether the self-assembly properties were dominant in plasma, or whether the fatty acid binding to human serum albumin (HSA) could compete for mPD5 oligomerization in plasma. To this end, we first measured the binding of mPD5 conjugated to Alexa Fluor 488 (mPD5-AF488) to HSA through FIDA (Figure 1I and Supplemental Figure 3) and found an affinity of mPD5-AF488 for HSA of 787 nM, which is 15-fold higher than the CMC (Figure 1F). *R*_H_ further suggests that mPD5 binds to dimeric HSA (Figure 1I). To evaluate whether mPD5 favors self-assembly or HSA binding in plasma, we incubated mPD5-AF488 in different concentrations of human plasma and obtained *R*_H_s suggesting binding to HSA is favored over micelle formation in plasma, even in a concentration of mPD5 (21 mM) that is 2000-fold above the CMC (Figure 1J and Supplemental Figure 4). Taken together, our data suggest that the lipid chain drives micellar assembly of mPD5, allowing for high solubility and good stability, as well as high-affinity PICK1 binding, whereas once in plasma, mPD5 preferentially binds to serum albumin at the given concentrations.

### mPD5 distributes to DRGs, but not the CNS.

**Figure 2:**
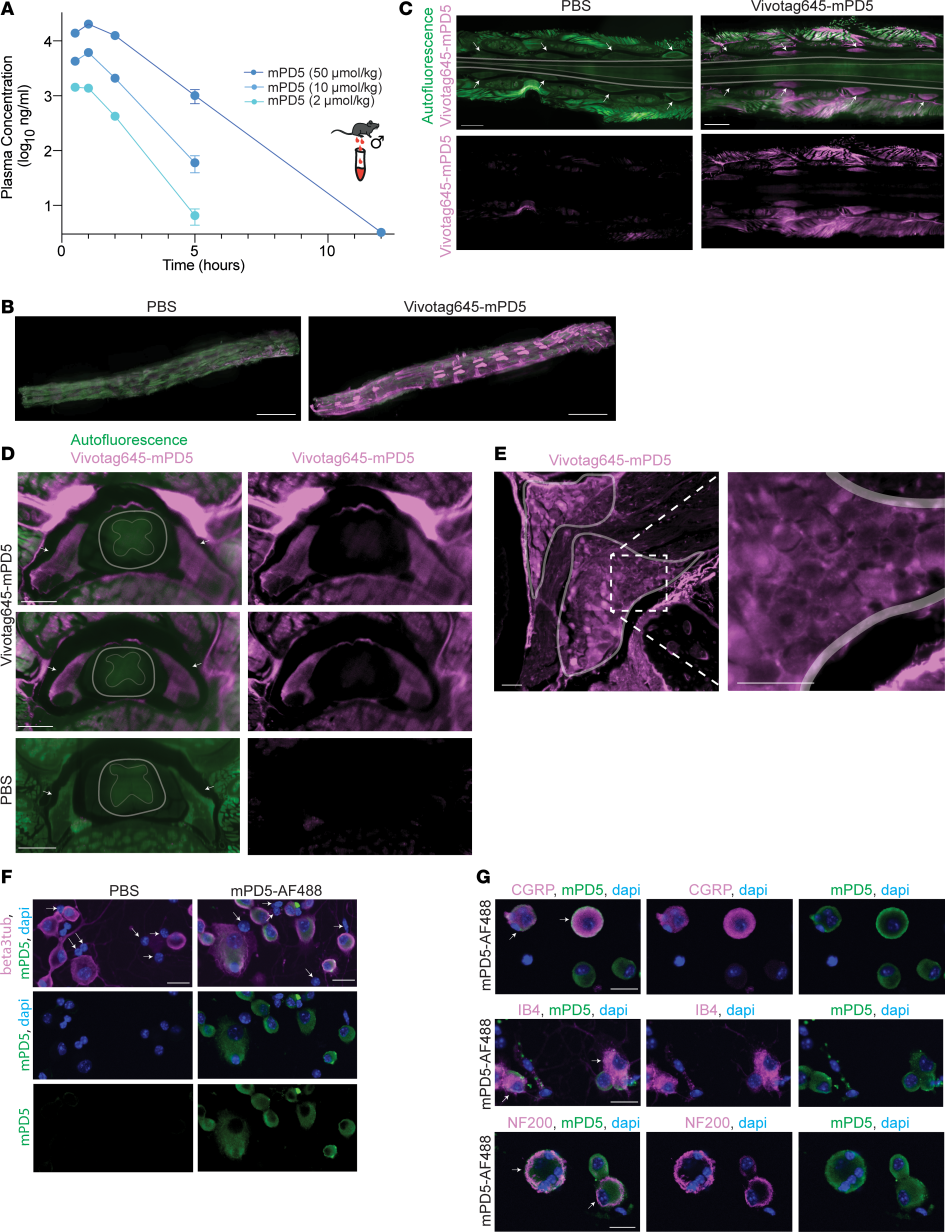
mPD5 distribution. (**A**) LC-MS/MS analysis of mPD5 plasma concentration at 0.5, 1, 2, 5, and 12 hours following s.c. injection in fasted male mice. Concentration peaked at 1 hour after injection in a dose-dependent manner (2 μmol/kg = 1.4 ± 0.1 mg/mL; 10 μmol/kg = 6.2 ± 5 mg/mL; 50 μmol/kg = 20.2 ± 0.6 mg/mL). mPD5 is eliminated with linear kinetics. *n* = 3. Lower limit of quantification = 2 ng/mL. (**B**) Maximum projection of 3D-imaged cleared spinal column with Vivotag645-mPD5 (magenta) and autofluorescence (green). Orientation: Caudal-rostral in the left-right direction and dorsal side facing up. Scale bars: 5000 μm. *n* = 3. (**C**) Optical section of 3D-imaged cleared lumbar spinal column in horizontal view with Vivotag645-mPD5 (magenta) and autofluorescence (green). Orientation: Caudal-rostral in the left-right direction at the level of the DRGs in the dorsal-ventral direction. Arrows point to DRGs. Scale bars: 1000 μm. *n* = 3. (**D**) Optical section of 3D-imaged cleared spinal column in transverse view with Vivotag645-mPD5 (magenta) and autofluorescence (green). Scale bars: 500 μm. Arrows point to DRGs. Gray area surrounds spinal column. *n* = 3. (**E**) Optical section of high-resolution light-sheet imaging of 1 DRG in cleared tissue. The 2 marked areas highlight regions with many neuronal cell bodies. The white dashed box indicates the magnified view. Scale bars: 50 μm. (**F**) Primary DRG culture stained against neurons for βIII-tubulin (magenta), mPD5-488 (green), and nuclei (blue). Arrows point to non-neuronal cells (all of which lack mPD5 signal). Scale bars: 20 μm. (**G**) Primary DRG culture stained against neuronal subtype markers CGRP, IB4, or NF200 (magenta), mPD5-488 (green), and nuclei (blue). Arrows point to double-positive cells for neuronal subtype marker and mPD5. Scale bars: 20 μm.

To assess pharmacokinetic properties of mPD5 and to guide dosing, we assessed dose dependence of the plasma exposure of mPD5 in mice following s.c. administration using a 5-fold descending dose range (50, 10, 2 μmol/kg) (Figure 2A). Plasma levels were assessed after 0.5, 1, 2, 5, and 12 hours. For all doses, we observed an initial increase in plasma concentration reaching maximal concentration after 1 hour (1.4 ± 0.1 mg/mL after 2 μmol/kg injection; 6.2 ± 5 mg/mL after 10 μmol/kg injection; 20.2 ± 0.6 mg/mL after 50 μmol/kg injection) followed by a linear elimination phase on the semi-log scale, indicating first-order kinetics. The maximal dose and area under the curve both scaled linearly with dose, and *t*_1/2_ showed a tendency to increase with increasing dose (0.50 ± 0.07 hours after 2 μmol/kg injection; 0.59 ± 0.07 hours after 10 μmol/kg injection; 0.84 ± 0.03 hours after 50 μmol/kg injection). For all concentrations, the distribution volume was approximately 30 mL, indicating good distribution to plasma from the site of injection.

To determine the distribution within the nervous system (Figure 2, B–E, and Supplemental Figure 5), PBS or mPD5 conjugated to VivoTag 645 (Vivotag645-mPD5, 10 μmol/kg) was injected s.c. 1 hour before transcardial perfusion followed by dissection of the brain and spinal column for whole-tissue clearing and light-sheet microscopy. Maximum projection of the Vivotag645-mPD5 signal showed distinct distribution within the spinal column (Figure 2B). Optical sectioning providing a horizontal view of the spinal column in the plane of the DRGs revealed distribution of Vivotag645-mPD5 to striated muscle surrounding the spinal column and to DRGs of the nervous system. Surprisingly, little if any signal was detected in the spinal cord, suggesting exclusion by the blood-brain barrier (Figure 2C). Transverse optical sectioning further supported this finding. While a clear signal (magenta) was evident in the DRGs (indicated by white arrows) of mice injected with Vivotag645-mPD5 (Figure 2D), no signal was observed in control mice (Figure 2D). For both groups, no signal was present within the spinal cord (white outline) (Figure 2D). Finally, the transverse view highlighted a high concentration of Vivotag645-mPD5 in patched structures on the dorsal part of the spinal canal (Figure 2D). Maximum projection of 3D-imaged cleared whole brains revealed Vivotag645-mPD5 signal in vascular and connective tissues (Supplemental Figure 5A), but with little if any signal within the brain tissue, as viewed in the sagittal plane, corroborating poor blood-brain-barrier penetrance (Supplemental Figure 5A). In accordance, we did not detect mPD5 in CSF, spinal cord, and brain tissue by mass spectrometry following s.c. injection of mPD5 in mice (Table 2). High-resolution light-sheet imaging of a single DRG suggested uptake of Vivotag645-mPD5 in somas of cells in the DRG (Figure 2E). To confirm cellular uptake in DRG neurons specifically, primary DRG cultures from adult mice were incubated with mPD5-AF488 (10 μM). Confocal imaging demonstrated cytosolic mPD5-AF488 signal surrounding the nuclei of neurons identified by βIII-tubulin staining, with no mPD5-AF488 signal in non-neuronal cells (DAPI positive, βIII-tubulin negative) (Figure 2F and Supplemental Figure 6A). Costaining with markers of neuronal subtypes did not indicate subtype selectivity (Figure 2G and Supplemental Figure 6B).

### Subcutaneous administration of mPD5 reduces mechanical and thermal hypersensitivity in a mouse model of inflammatory pain.

**Figure 3:**
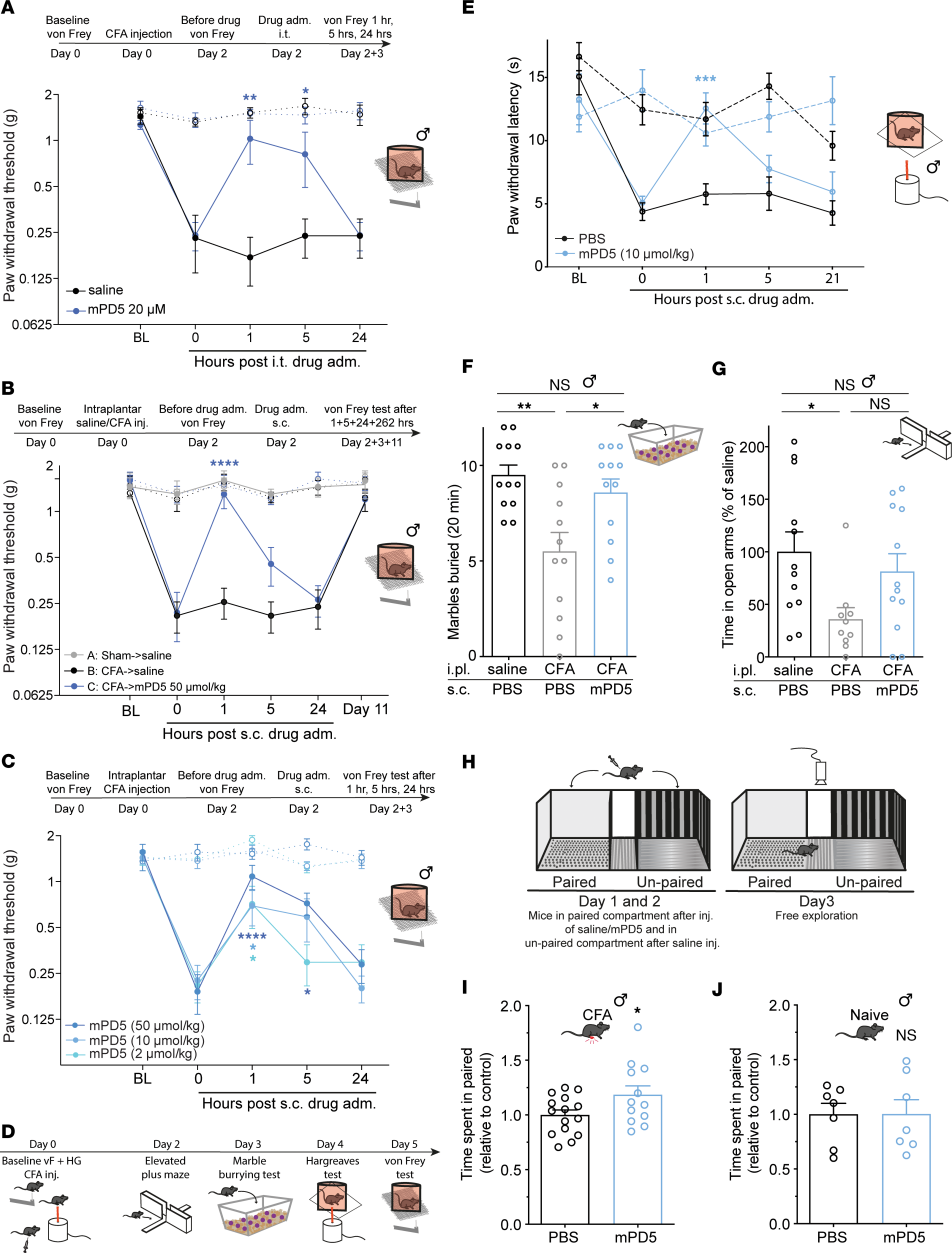
Efficacy of mPD5 in a mouse model of inflammatory pain. (**A**) Paw withdrawal threshold (PWT) before and after induction of inflammatory pain (CFA-induced) and treatment (i.t.) with mPD5 or saline. *n*_saline_= 5, *n*_mPD5_ = 6. (**B**) PWT before and after induction of inflammatory pain or sham and s.c. treatment with mPD5 or saline. *n*_saline→saline_ = 4, *n*_CFA→saline_ = 5, *n*_CFA→mPD5_ = 6. (**C**) PWT before and after induction of inflammatory pain and s.c. treatment with mPD5. *n*_50_ = 5, *n*_10_ = 6, *n*_2_ = 5. (**D**) Timeline of **E**–**G**. (**E**) Paw withdrawal latency before and after induction of inflammatory pain and s.c. treatment with mPD5 or PBS. *n* = 6. (**F**) Marbles buried after induction of inflammatory pain or sham 1 hour after s.c. treatment with mPD5 or PBS. *n* = 12. (**G**) Time spent in open arms of an elevated plus maze relative to sham, after induction of inflammatory pain or sham 1 hour after s.c. treatment with mPD5 or PBS. *n* = 12. (**H**) Schematic overview of sePP with 2 counterbalanced days of conditioning followed by a test day. (**I**) Time spent in the paired compartment of mice in inflammatory pain conditioned with 30 μmol/kg mPD5 or saline in the paired compartment. *n*_PBS_= 13, *n*_mPD5_ = 12. (**J**) Time spent in the paired compartment of naive mice conditioned with 30 μmol/kg mPD5 or saline in the paired compartment. *n* = 7. adm., administration; BL, baseline; CFA, complete Freund’s adjuvant; HG, Hargreaves test; hrs, hours; inj., injection; i.pl., intraplantar; i.t., intrathecal; s.c., subcutaneous; sePP, single exposure place preference; NS, not significant. Dashed lines in **A**–**C** and **E** indicate the contralateral paw. *P < 0.05; **P < 0.01; ***P < 0.001; ****P < 0.0001 by 2-way ANOVA with Dunnett’s post hoc test vs. 0 hours (**A**–**C** and **E**), 1-way ANOVA with Tukey’s test (**F** and **G**), or unpaired, 2-tailed *t* test (**I** and **J**).

We tested the effect of i.t. administered mPD5, similar to what was done with TPD5 (8), using the complete Freund’s adjuvant (CFA) model of inflammatory pain (Figure 3A). The experiment was performed as follows: on day 0, the baseline mechanical paw withdrawal threshold (PWT) was established before intraplantar injection with CFA, which gives rise to a behavioral indication of allodynia reverting after 11 days, consistent with previous studies (20, 40) (Figure 3, A and B). Mice injected with saline instead of CFA (sham) showed no changes in their PWT (Figure 3B). On day 2 after CFA injection and following randomization, mice were injected with mPD5 (i.t., 20 μM, 7 μL), resulting in a significant relief of mechanical hypersensitivity at 1 hour and 5 hours after administration, with no effect 24 hours after administration (Figure 3A). The efficacy of mPD5 by this route of administration was very similar to that of TPD5 (confirming effects on spinal transmission), as was duration of action, albeit with slightly faster onset kinetics (8).

Encouraged by the biodistribution followed by s.c. administration (Figure 2), we tested the effect of mPD5 given by this route of administration in the CFA model of inflammatory pain in male mice (Figure 3, B–J). A single s.c. administered high dose (50 μmol/kg) of mPD5 reverted hypersensitivity 1 hour after injection compared with before injection (Figure 3B). Next, we tested the dose dependence of mPD5 in the CFA model using the doses tested for plasma concentrations (s.c., 2, 10, and 50 μmol/kg, 10 μL/g) (Figure 3C). All doses tested revealed significant reversion of mechanical hypersensitivity 1 hour after administration, and the 50 μmol/kg dose also showed significant reduction of mechanical hypersensitivity 5 hours after injection. In combination with the exposure data (Figure 2A), this indicates that a plasma concentration of approximately 1 mg/mL is sufficient to evoke a significant reduction in mechanical hypersensitivity. The ability of mPD5 to revert CFA-induced mechanical hypersensitivity was confirmed in female mice (s.c., 10 μmol/kg) (Supplemental Figure 7A).

To test the effect of mPD5 on a different pain-related sensory modality, we assessed thermally evoked hypersensitivity in the CFA model using the Hargreaves test (Figure 3E). Following baseline testing, mice were injected with CFA and randomly assigned into treatment versus vehicle groups. Intraplantar injection of CFA into the hind paw led to thermal hypersensitivity of the ipsilateral paw, a behavioral indication of thermal hyperalgesia, which was reverted by mPD5 (s.c., 10 μmol/kg) 1 hour after administration. No effect of either CFA or mPD5 was observed on the contralateral paw (Figure 3E).

### mPD5 reduces pain-related behaviors in a mouse model of inflammatory pain.

In the clinic, several comorbidities of chronic pain have been identified, including anxiety, depression, and fatigue (41). Mechanically and thermally evoked hypersensitivity do not assess aspects of ongoing pain, functional impairment, nor anxiodepressive symptoms associated with pain, all of which are clinically important pain-related symptoms (42). Therefore, we used the combination of a marble-burying test, elevated plus maze, and single exposure place preference (sePP) to qualify the therapeutic relevance of mPD5 (Figure 3, D and F–J). In the marble-burying test (Figure 3F), CFA injection led to significantly decreased marble burying, presumably anxiogenic. This decrease was reverted significantly by treatment with 10 μmol/kg mPD5 to the level of the naive mice. In the elevated plus maze test (Figure 3G), CFA injection led to significantly decreased time spent in the open arms, presumably also reflecting an anxiogenic effect. Following mPD5 treatment (s.c., 10 μmol/kg) of the CFA-injected animals, the amount of time spent in the open arms was no longer significantly different from the naive mice. Finally, we used a sePP setup (43) to estimate the initial perception of the drug as a measure of relief of ongoing pain (Figure 3, H–J). The experiment was performed in a 3-compartment apparatus with a striped and a gray compartment separated by a neutral zone (Figure 3H). CFA animals were injected with either PBS in both compartments or mPD5 (s.c., 30 μmol/kg) in the gray (paired) compartment and PBS in the striped (unpaired) compartment. Interestingly, the CFA animals treated with mPD5 spent significantly more time in the paired compartment compared with the PBS animals, indicating a positive effect of mPD5 on spontaneous/ongoing pain (Figure 3I). Due to the high abuse liability of the current chronic pain treatments (44), we repeated the experiment on naive mice to assess putative intrinsic rewarding properties of mPD5 (Figure 3J). The initial sensitivity to the rewarding properties of drugs is believed to be an important endophenotype in relation to the vulnerability to addiction and the initial sensitivity to the rewarding properties of a specific drug is a relevant indicator of addictive properties of said drug (43, 45). Importantly, such control animals did not show any preference for mPD5 compared to PBS (Figure 3J), indicating no intrinsic rewarding properties of mPD5.

### mPD5 reduces mechanical hypersensitivity in mice following SNI and streptozocin injection, but not cancer-induced bone pain.

**Figure 4:**
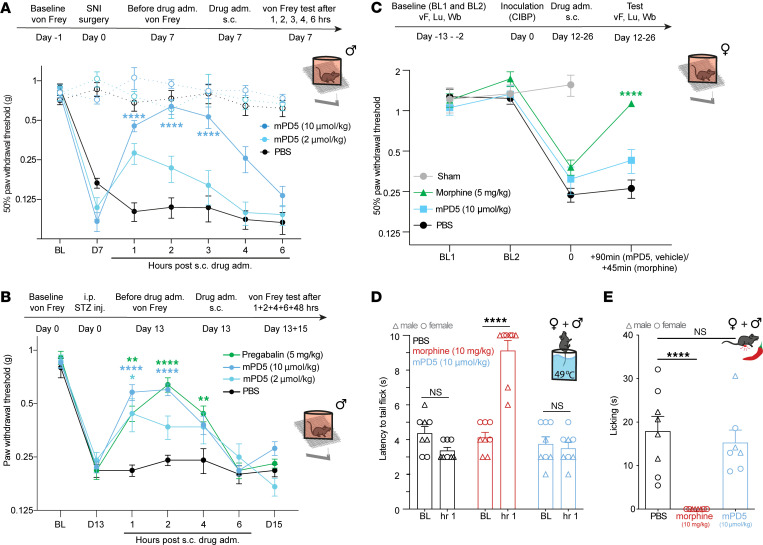
Efficacy of mPD5 in neuropathic pain, diabetic neuropathy, bone cancer–induced pain, and acute nociception. (**A**) Paw withdrawal threshold (PWT) before and after induction of neuropathic pain (SNI surgery) and s.c. treatment with mPD5 or PBS 7 days after surgery. *n* = 6 in each group. Dashed line indicates the contralateral paw. (**B**) PWT before and after induction of diabetic neuropathy (STZ injection) and s.c. treatment with mPD5, pregabalin, or PBS 13 days after STZ injection. *n*_pregabalin_ = 10, *n*_mPD5_ = 10, and *n*_PBS_ = 9. (**C**) PWT before and after induction of cancer-induced bone pain (sham or NCTC 2472 cell inoculation) and s.c. treatment with mPD5, morphine, or PBS. *n*_sham_ = 5, *n*_morphine_ = 10, *n*_mPD5_ = 11, and *n*_PBS_ = 11. (**D** and **E**) Efficacy of mPD5 on acute pain in female (o) and male (Δ) mice. (**D**) Effect of PBS, morphine (10 mg/kg), and mPD5 (10 μmol/kg) on tail-flick time in water of 49°C ± 0.5°C. *n* = 8. (**E**) Effect of PBS, morphine (10 mg/kg), and mPD5 (10 μmol/kg) on capsaicin-induced licking time. *n*_morphine_ = 8, *n*_mPD5_ = 7, and *n*_PBS_ = 8. adm., administration; BL, baseline; CIBP, cancer-induced bone pain; D, day; hrs, hours; inj., injection; NS, not significant; s.c., subcutaneous; SNI, spared nerve injury; STZ, streptozocin. *P < 0.05; **P < 0.01; ***P < 0.001; ****P < 0.0001 by 2-way ANOVA with Dunnett’s post hoc test vs. 0 hours (**A**–**C**), 2-way ANOVA with Šidák’s post hoc test of BL vs. 1 hour (**D**), or 1-way ANOVA with Dunnett’s post hoc test of PBS vs. drug (**E**).

We next evaluated the pain-relieving effects of mPD5 in different neuropathic pain models. First, we investigated the effect of mPD5 in the SNI model in male mice (Figure 4A). On day 0, the baseline mechanical PWT was established, followed by SNI surgery. SNI surgery led to a significant decrease in PWT on day 7 versus baseline, a behavioral indication of mechanical allodynia. We found that mPD5 (s.c., 10 μmol/kg) significantly reduced mechanical hypersensitivity up to 3 hours, whereas a lower dose (s.c., 2 μmol/kg) showed no significant effect, indicating slightly lower potency in the SNI model as compared with the CFA model. Next, we assessed the treatment efficacy of mPD5 to relieve diabetic neuropathy using the streptozocin (STZ) model of type 1 diabetes in male mice (Figure 4B). On day 0, the baseline mechanical PWT was established, followed by injection of STZ. On day 7 after STZ injection, all mice presented a drastic increase in glycemia (from 197.4 ± 4.4 to 533.5 ± 10.4 mg/dL), validating the diabetic state of the mice. STZ injection led to a significant decrease in PWT on day 13 versus baseline, a behavioral indication of mechanical allodynia (Figure 4B). Since the STZ model affects both paws equally, we used the established pain-relieving drug pregabalin as a positive control in the experiment instead of the contralateral paw. mPD5 (s.c., 2 and 10 μmol/kg) resulted in a significant relief of mechanical hypersensitivity the first hour after injection, and this effect was extended for another hour at the highest dose. The positive control (pregabalin) resulted in significant pain relief up to 4 hours after injection, whereas the vehicle (PBS) had no effect on mechanical hypersensitivity.

Finally, we assessed the effect of mPD5 in a cancer-induced bone pain (CIBP) model (Figure 4C and Supplemental Figure 7, B and C). Female mice were inoculated with a sarcoma cell line (NCTC 2472) in the femur marrow cavity. Pain-like behavior was assessed every second day until meeting the criteria of a limb-use score of 2 or below in combination with a weight-bearing ratio of 0.35 or below, where they received treatment with mPD5 (s.c. 10 μmol/kg) or vehicle. Mice met the criteria at 12–26 days after inoculation. Since symptoms of pain are notoriously challenging to relieve in CIBP models, we used morphine as a positive control. Indeed, morphine (s.c., 5 mg/kg) gave rise to a partial but significant increase in PWT, limb-use score, and weight-bearing ratio in the animals, whereas mPD5 and vehicle had no effect on either.

### mPD5 has no effect on nociceptive responses in naive mice.

We next assessed the effect of mPD5 in 2 models of acute pain in both sexes. In the hot water tail immersion test, morphine (s.c., 10 mg/kg) significantly reduced the latency to tail-flick compared with baseline, whereas PBS and mPD5 (s.c., 10 μmol/kg) had no effect on tail-flick latency (Figure 4D). Similarly, morphine (s.c., 10 mg/kg) significantly reduced the licking time of mice following intraplantar (i.pl.) injection of capsaicin, whereas PBS and mPD5 (s.c., 10 μmol/kg) had no effect on time spent licking (Figure 4E).

### mPD5 reduces pain-related behaviors in a mouse model of neuropathic pain.

**Figure 5:**
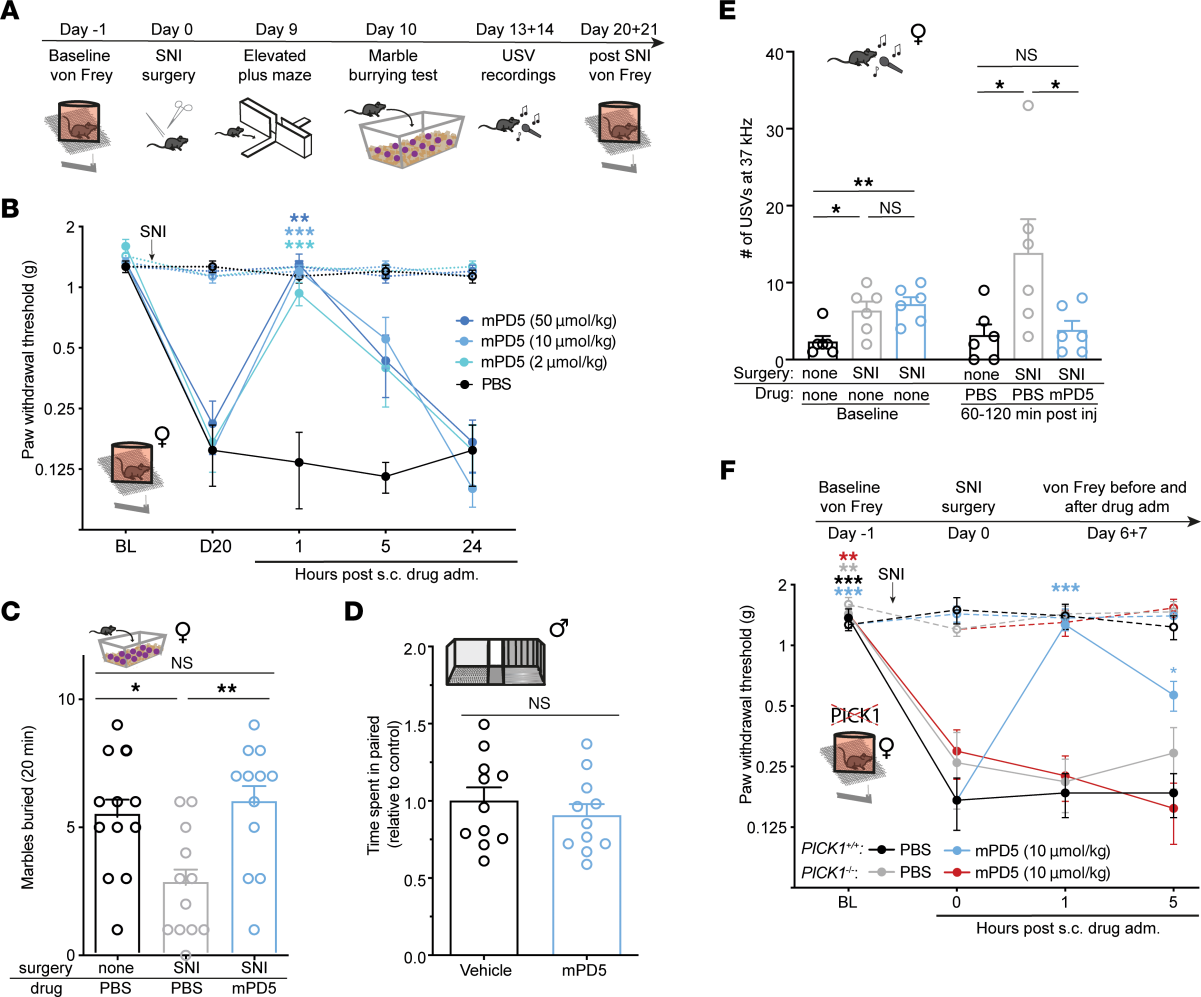
Efficacy of mPD5 in a model of neuropathic pain in WT and PICK1-KO mice. (**A**) Timeline for **B**, **C**, and **E**. (**B**) Paw withdrawal threshold (PWT) before and after induction of neuropathic pain (SNI surgery) and s.c. treatment with mPD5 or PBS. *n* = 6. (**C**) Marbles buried after induction of neuropathic pain or naive 1 hour after s.c. treatment with mPD5 or PBS. *n* = 12 in each group. (**D**) Time spent in the paired compartment of SNI mice conditioned with 30 μmol/kg mPD5 or saline in the paired compartment. *n* = 11. (**E**) Recordings of ultrasonic vocalizations of SNI and naive mice at 37 kHz made for 60 minutes at baseline and 60–120 minutes after s.c. treatment with mPD5 or PBS. *n* = 6. (**F**) PWT before and after induction of neuropathic pain and s.c. treatment with mPD5 or PBS in PICK1-WT and -KO mice. Cross-sectional study ending up with *n* = 6 in each group. adm., administration; BL, baseline; D, day; inj., injection; NS, not significant; s.c., subcutaneous; SNI, spared nerve injury; USV, ultrasonic vocalizations. *P < 0.05; **P < 0.01; ***P < 0.001 by 2-way ANOVA with Dunnett’s post hoc test vs. 0 hours (**B** and **F**), 1-way ANOVA with Dunnett’s multiple-comparison test (**C**), unpaired, 2-tailed *t* test (**D**), or 1-way ANOVA with Dunnett’s post hoc test of SNI-PBS vs. the other 2 groups at baseline and after treatment (**E**). In **B** and **F**, the dashed line indicates the contralateral paw.

To further explore the effect of mPD5 on neuropathic pain, we returned to the combination of von Frey, marble-burying test, and elevated plus maze, this time in female mice using the SNI model (Figure 5A). Using von Frey, we found that mPD5 significantly reduced mechanical hypersensitivity 1 hour after injection at all tested doses (s.c., 2, 10, 50 μmol/kg), with no effect of PBS (Figure 5B). In the marble-burying test (Figure 5C), SNI surgery led to significantly decreased marble burying, and this decrease was reverted significantly by mPD5 treatment (s.c., 10 μmol/kg) to the level of the naive mice. In the elevated plus maze, the SNI surgery did not affect the time spent in open arms compared to naive mice (Supplemental Figure 7D). We tested the effect of mPD5 in the sePP for the SNI model (Figure 5D) using the same setup as for the CFA model (Figure 3H). However, mPD5 (s.c., 30 μmol/kg) did not change the time spent in the paired chamber compared to PBS in the SNI model (Figure 5D). Only males were used in this experiment, since it is known that the initial perception of the drug is not sufficient to alter place preference in females for all drugs (43). As an alternative measure of the ongoing pain perception, we recorded ultrasonic vocalizations of the female mice. SNI surgery led to significantly more vocalizations at 37 kHz, and this increase was fully reversed by mPD5 treatment (s.c., 10 μmol/kg) to the level of the naive mice (Figure 5E).

### mPD5 does not revert hypersensitivity following SNI surgery in mice lacking PICK1.

It has previously been shown that the hypersensitivity of PICK1-KO mice is significantly blunted in the L5 spinal nerve ligation model (21), in which transection is made close to the DRG of the L5 only (46). However, this difference was not significant in female PICK1-KO mice following SNI surgery (Figure 5F), in which the common peroneal and tibial nerves are cut distal to the DRGs of the L3–L5 (46). This indicates that the plasticity induced by the 2 models (47) may differentially rely on PICK1. Nevertheless, treatment with mPD5 had no effect on mechanical hypersensitivity in PICK1-KO mice following SNI surgery, whereas it was significantly reduced in WT littermates, consistent with PICK1 being the target of mPD5 (Figure 5F).

### mPD5 does not affect general locomotion, fertility, or learning and memory.

**Figure 6:**
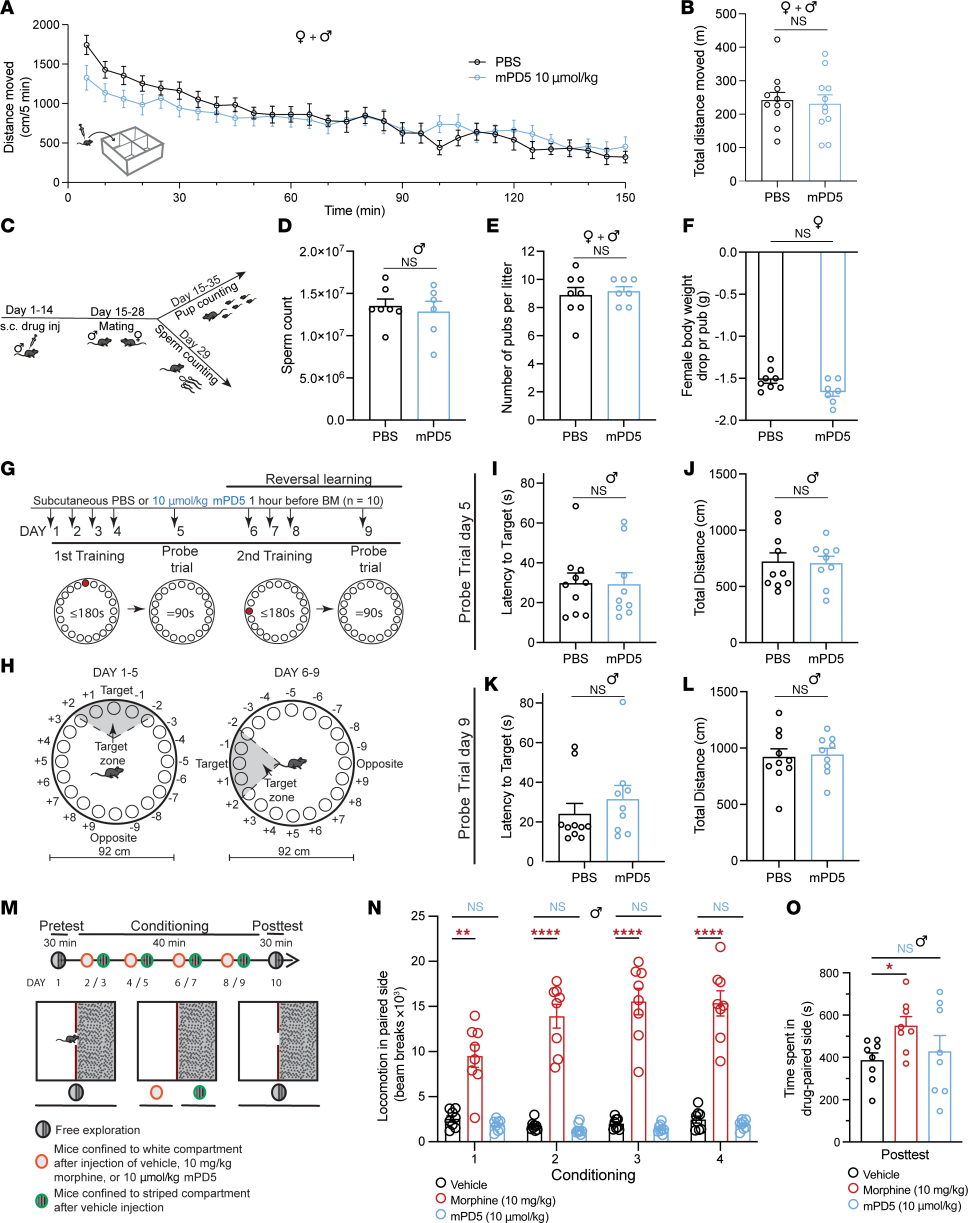
Effect of mPD5 on naive animals. (**A** and **B**) Locomotor response of mice injected s.c. with PBS or 10 μmol/kg mPD5 and left in open field boxes (white, 40 × 40 × 80 cm) for 150 minutes. Data are depicted in bins of 5 minutes (**A**) and as total locomotion (**B**). (*n* = 11 [5 female, 6 male]). (**C**) Timeline of fertility study shown in **D**–**F**. (**D**) Sperm count (*n*_PBS_ = 7, *n*_mPD5_ = 6). (**E**) Number of pups per litter (*n*_PBS_ = 8, *n*_mPD5_ = 7). (**F**) Female body weight difference before and after labor divided by number of pups (*n*_PBS_ = 8, *n*_mPD5_ = 7). (**G**–**L**) Effect of mPD5 on Barnes maze performance. (**G**) Schematic overview of the Barnes maze experiment, including 3 days of reversal learning (*n*_PBS_ = 10, *n*_mPD5_ = 9). (**H**) Schematic illustration of the Barnes maze used. (**I** and **J**) Probe test on day 5 following initial 4 days of training. (**I**) Latency to reach target hole. (**J**) Total distance moved. (**K** and **L**) Probe test on day 9 following 3 days of reversal learning. (**K**) Latency to reach target hole. (**L**) Total distance moved. (**M**–**O**) Conditioned place preference (CPP). (**M**) Schematic overview of CPP (*n* = 8). (**N**) Total locomotion of the 3 groups in the drug-paired compartment for the 4 days of conditioning in the white side of the CPP apparatus. (**O**) Time spent in the drug-paired compartment following conditioning. BM, Barnes maze; inj., injection; NS, not significant; s.c., subcutaneous. *P < 0.05; **P < 0.01; ****P < 0.0001 by 2-way ANOVA with Šidák’s multiple-comparison test (**A**), 1-way ANOVA with uncorrected Fischer’s LSD (**B** and **O**), unpaired, 2-tailed *t* test (**I**–**L**), or 2-way ANOVA with Dunnett’s post hoc test (**N**).

Drug treatments of chronic pain are compromised by dose-limiting side effects (48, 49). To assess potential generalized side effects in terms of motor function, sedation, and hyperactivity, mice of both sexes were injected with PBS or mPD5 (s.c.,10 μmol/kg) and placed in an open field for 150 minutes (Figure 6, A and B). A tendency toward lower locomotion was observed initially (although not significant at any individual time bin), and the overall locomotion was the same between groups. Mice injected with higher doses (s.c., 30 or 50 μmol/kg) of mPD5 also did not show any significant effect on locomotor activity (Supplemental Figure 8A).

Next, to address putative on-target side effects, we tested the effect of repeated administration of mPD5 (s.c., 10 μmol/kg, once daily for 14 days) versus vehicle on male fertility (Figure 6, C–F, and Supplemental Figure 8, B and C), since complete loss of PICK1 is known to cause male infertility (50). The sperm count of the males was the same between groups (Figure 6D), as was the number of pups per litter (Figure 6E and Supplemental Figure 8B). Although the weight gain was significantly higher for the females mated with mPD5-treated male mice (indicating heavier litter) (Supplemental Figure 8C), the body weight drop per pup following birth was the same for both groups (Figure 6F).

In the CNS, PICK1 has been strongly implicated in synaptic plasticity underlying learning and memory, as well as addictive processes (12, 18). Consequently, despite the apparent exclusion of the peptide from the CNS (Figure 2), we tested the effect of mPD5 on Barnes maze performance (Figure 6, G–L) as well as conditioned place preference (CPP) (Figure 6, M–O). For the Barnes maze experiment, mice were injected with PBS or mPD5 (s.c., 10 μmol/kg) 60 minutes before placement on the maze. Following 4 days of training (and 4 injections in total), the 2 groups showed no difference in latency to reach target (Figure 6I) or total distance moved (Figure 6J), indicating no effect on learning or recall. Likewise, no difference was observed in latency to reach the target (Figure 6K) or total distance moved (Figure 6L) following reversal learning. The overall distribution of nose pokes between the different holes also did not differ between groups in either test (Supplemental Figure 8, D and E).

### mPD5 does not show abuse liability.

Finally, current chronic pain treatments, and opioids in particular, show high abuse liability (6). To compare the abuse liability of mPD5 to morphine, we performed a CPP in naive animals (Figure 6M). Morphine significantly increased locomotion during conditioning compared with PBS, whereas mPD5 did not affect locomotion in either direction (Figure 6N). As anticipated, the group treated with morphine spent significantly more time in the drug-paired compartment during the posttest compared with the PBS group, while the mPD5 group did not (Figure 6O).

### mPD5 provides sustained relief of mechanical hypersensitivity in the chronic phase of neuropathic pain.

**Figure 7:**
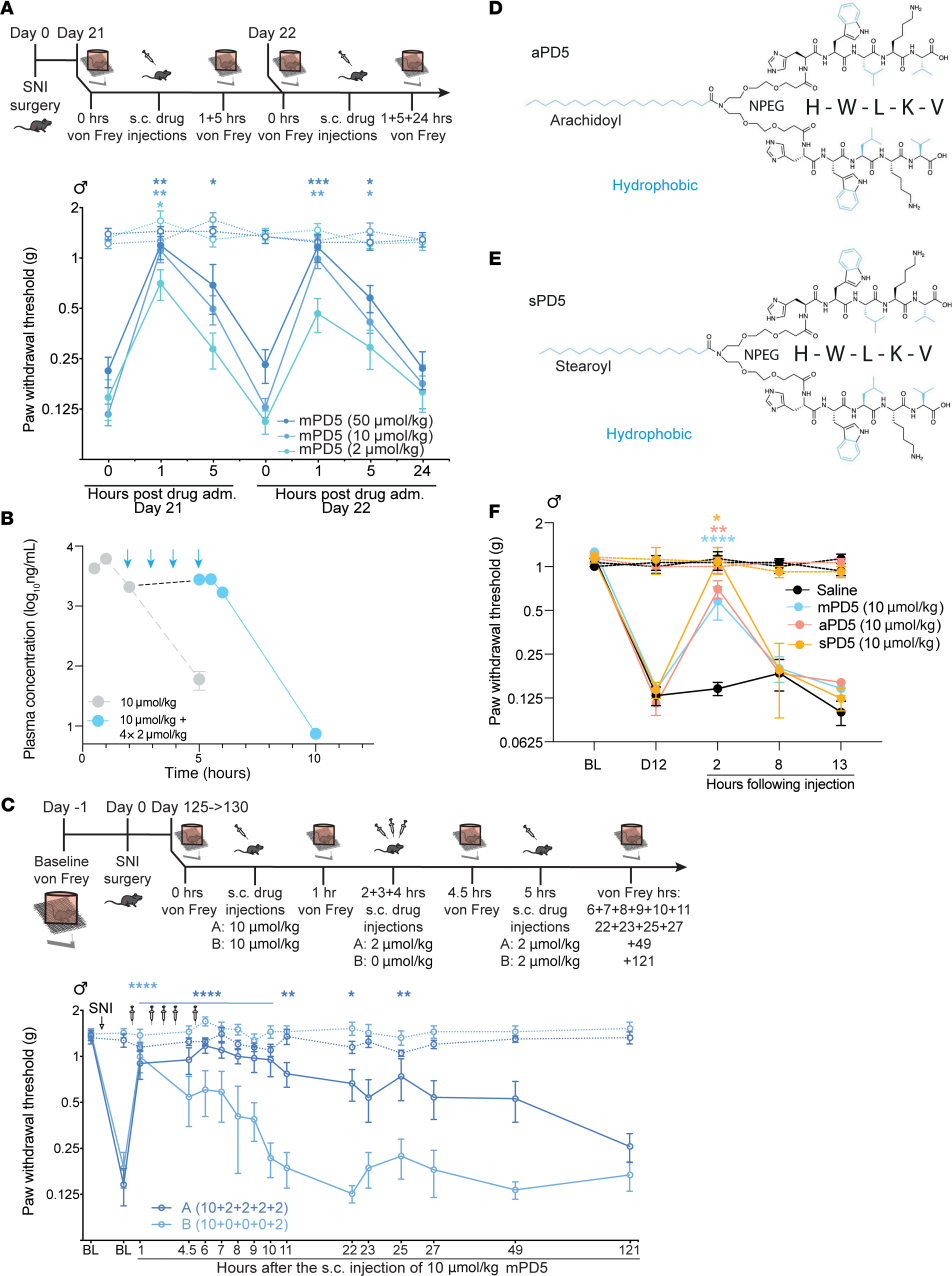
Efficacy of mPD5 on late-stage neuropathic pain following sustained dosing. (**A**) Paw withdrawal threshold (PWT) before and after induction of neuropathic pain (SNI surgery) and repeated s.c. treatment with mPD5. *n* = 8 in each group. (**B**) Plasma concentration of mPD5 (blue) determined by LC-MS/MS in mice injected s.c. with 10 μmol/kg mPD5 followed by 2 + 2 + 2 + 2 μmol/kg mPD5 once an hour. mPD5 was eliminated with linear kinetics similar to the single administration (gray, dashed line, identical to Figure 2A), shown again for comparison. (**C**) PWT before and after induction of SNI and sustained s.c. treatment with 10 + 2 + 2+ 2 + 2 μmol/kg or 10 + 0 + 0 + 0 + 2 μmol/kg (1 hour between injections) mPD5. *n* = 8. (**D**) Structure of arachidoyl-NPEG_4_-(HWLKV)_2_ (peptide conjugated with C_20_ [arachidic acid]) (aPD5) and (**E**) stearoyl-NPEG_4_-(HWLKV)_2_ (peptide conjugated with C_18_ [stearic acid]) (sPD5). (**F**) PWT before and after SNI surgery and s.c. treatment with saline, aPD5, sPD5, or mPD5. *n* = 6. adm., administration; BL, baseline; D, day; hrs, hours; inj., injection; s.c., subcutaneous; SNI, spared nerve injury. In **A**, **C**, and **F**, the dashed line indicates the contralateral paw. *P < 0.05; **P < 0.01; ***P < 0.001; ****P < 0.0001 by 2-way ANOVA with Dunnett’s post hoc test (**A**, **C**, and **F**).

To test for putative development of tolerance due to downregulation of the target or other adaptive mechanisms, we carried out a full dose dependence of mPD5 in the SNI model (3 weeks after surgery) in mice on 2 consecutive days (s.c., 2, 10, 50 μmol/kg mPD5) (Figure 7A). We observed dose-dependent relief of the mechanical hypersensitivity for all concentrations tested, giving rise to significant effects at 1 hour, while only the 50 μmol/kg dose showed a significant effect at 5 hours. Such dose dependency was also evident the next day. Here, only the 2 highest concentrations showed significant pain relief, but now at both 1 and 5 hours after administration. Taken together, these results do not indicate immediate development of tolerance.

With this in mind, we sought to obtain sustained relief of mechanical hypersensitivity by consecutive administrations of mPD5. To estimate dose and dosing interval for maintaining a steady-state plasma concentration, we employed van Rossum’s equation using parameters obtained from the single administration (*D* = 10 μmol/kg, Vd ~30 mL, *t*_1/2_ = 0.6 hours) (Figure 2A). This predicted that a steady-state plasma concentration of approximately 2 mg/mL, following a 10 μmol/kg bolus injection, could be maintained by a dosing of 2 μmol/kg administered once an hour. We experimentally verified this prediction by assessing the plasma exposure following a single s.c. injection of 10 μmol/kg mPD5 followed by 4 consecutive administrations of 2 μmol/kg in 1-hour intervals (Figure 7B). Blood samples were taken at 5 minutes, 30 minutes, 1 hour, 5 hours, 12 hours, 24 hours, and 48 hours after the last administration. Notably, the level immediately after the last injection was 2.8 ± 0.5 mg/mL, in good agreement with model prediction. The subsequent kinetics were comparable to the single administration, with a *t*_1/2_ of 0.52 ± 0.02 hours.

Next, we assessed how a repeated administration paradigm giving rise to a steady-state plasma level of the drug would affect mechanical hypersensitivity in the SNI model of chronic neuropathic pain. To this end, we used mice in a very late stage of the SNI model (18 weeks after surgery), where mice were injected with either 10 + 2 + 2 + 2 + 2 μmol/kg mPD5 (Group A) or 10 + 0 + 0 + 0 + 2 μmol/kg mPD5 as reference (Group B) (1 hour between injections) (Figure 7C). Such consecutive administration of mPD5 not only showed sustained relief of mechanical hypersensitivity, but surprisingly also extended the duration of effect from 1 hour to 25 hours (20 hours following last injection). Finally, we asked whether such extended duration of action could be obtained by using peptides where the myristic acid was replaced by longer acyl chains to increase their plasma lifetimes (51). For this, we tested peptides conjugated with C_18_ (stearic acid) or C_20_ (arachidic acid) fatty acids to produce sPD5 and aPD5, respectively (Figure 7, D–F). Single s.c. administration of sPD5 and aPD5 both caused profound itching in the mice, but nonetheless relieved mechanical hypersensitivity in the SNI model, similar to mPD5 2 hours after injection. Similar to mPD5, however, there was no effect on mechanical hypersensitivity 8 hours after injection, suggesting that the extended effect could not be achieved by increasing the length of the fatty acyl chain only.

## Discussion

In this study, we have shown that mPD5 relieved pain in 3 different pain models with fundamentally different etiologies: the CFA model of acute inflammatory pain as well as the SNI and STZ models of chronic neuropathic pain. We also report that mPD5 showed no effect in a CIBP model, where pain is known to be very challenging to treat (52). Importantly, a number of changes occur in the DRGs, as well as the dorsal horn following induction of both inflammatory and neuropathic pain models (53). In murine models of inflammatory, neuropathic, and cancer pain it has been shown that each model generated a unique set of neurochemical changes in the spinal cord and sensory neurons (54). In C3H/Hej mice, changes in substance P and calcitonin gene–related peptide are observed in models of inflammatory (CFA) and neuropathic (sciatic nerve transection or L5 spinal nerve ligation) pain models, with no changes in levels of substance P and calcitonin gene–related peptide observed in the model of cancer pain (injection of osteolytic sarcoma cells into the femur), suggesting that cancer induces a unique, persistent pain state (54). The full efficacy of mPD5 in neuropathic pain combined with complete lack of efficacy in CIBP suggests that these models modify nociceptive transmission differently based on their dependence on PICK1.

We recently published on a TAT-conjugated, bivalent, high-affinity PICK1 inhibitor (TPD5) displaying robust efficacy in the SNI model of neuropathic pain and CFA model of inflammatory pain following i.t. administration in mice (8, 22). Efficacy of TPD5, however, was relatively low following systemic administration (i.p. and s.c.) and increased dosing caused significant discomfort of the mice. Symptoms included itching, respiratory abnormality, and immobility, which has been reported also for another TAT-conjugated cell-permeable peptide (TAT NR2B9c, US patent 8,080,518 B2). Of additional concern, the TAT sequence itself has been shown to alter the expression of specific genes (both induction and repression) in HeLa cells (35). In the current paper, to circumvent potential safety issues with the TAT cell-penetrating peptide, we developed mPD5, which was obtained by substitution of TAT with a C_14_ fatty acid (myristic acid). Myristoylation of simple peptides to modulate synaptic transmission and plasticity has been used previously and render them cell permeable (7, 55, 56). In drug development, lipidation of peptides has been employed to enhance plasma stability due to the interaction between lipids and serum albumin (32, 33, 57, 58). Studies suggested that lipidation allowed the formation of higher-order structures (59–61), which increased the solubility and resilience to degradation. Presumably, mPD5 benefits from all these consequences of the myristoylation, and the compound relies on well-known chemical principles and well-tested building blocks that are considered safe in humans (33, 62). In our case, the change from TPD5 to mPD5 turned out advantageous since it allowed for efficacious s.c. administration. Subcutaneous administration of peptidic drugs is more advantageous than intravenous or i.t. administration in terms of improving patient compliance, e.g., due to the suitability for self-administration (63). Subcutaneous injection is further valued due to the avoidance of hepatic and gastrointestinal degradation. Clinically, s.c. injection is the most common route of administration for peptides and is used extensively for both continuous and low-dose drug treatment (63–65).

It has been hypothesized that the poor translational value of preclinical data of rodents to humans is because assessment of hypersensitivity using von Frey and Hargreaves relies on withdrawal reflexes and thus should not stand alone (66). Our data showed that animals with inflammatory pain preferred the mPD5-paired compartment over vehicle treatment in the sePP test, indicating that the effect of mPD5 is not merely reflex inhibition. Moreover, mPD5 reduced anxiodepressive behaviors associated with pain in both inflammatory and neuropathic models, as well as reduced ultrasonic vocalizations of mice in neuropathic pain. This suggests that the mechanism of mPD5 indeed taps into the complex pattern of symptoms that are of relevance in patients with chronic pain.

Many drugs used in the treatment of chronic pain show highly problematic central side effects, including sedation, confusion, and memory problems (67, 68). Unlike opioids (67) and gabapentinoids (68), mPD5 did not significantly affect novelty-induced exploration, general locomotor activity, or memory and learning. For opioids in particular there is a high risk of substance abuse (69), but abuse liability of gabapentinoids is also gaining attention (44). Indeed, morphine conferred CPP in our assessment, while the preference for the mPD5-paired compartment was not different from vehicle following both single and multiple exposures to mPD5. In combination with the data indicating that mPD5 does not reach the CNS, these experiments collectively argue that the potential abuse liability of mPD5 is low. However, despite the lack of effect on the mean preference change in both experiments (Figure 3J and Figure 6O), it does seem from both experiments that the mPD5 group potentially splits into 2 groups. Together with the tendency of reduced locomotion (nonsignificant) in the exploratory phase of the open field test and our c-Fos data in obese mice (70), this might warrant further studies of (indirect) effects of mPD5 on the dopamine system. Finally, in contrast with other peripherally acting drugs, such as lidocaine, mPD5 relieved maladaptive pain specifically while retaining acute nociceptive and mechanical sensation. Relieving chronic pain, without limiting the sensitivity to potential harmful stimuli of everyday life (unlike, i.e., morphine), would be a great benefit for patients.

In conclusion, we have shown that mPD5 relieved ongoing and evoked hypersensitivity in multiple mouse models of pain in female and male mice with cross-laboratory validation for the SNI model. mPD5 displayed favorable pharmacokinetic properties (easily soluble and highly stable). It alleviated evoked pain (thermal and mechanic) following different routes of administration (i.t. and s.c.), in inflammatory (CFA) and neuropathic pain models (SNI and STZ) and was efficacious in transient and chronic pain. Notably, and important for the translational potential of mPD5, it also reduced anxiodepressive behavior (marble-burying test and elevated plus maze) and pain-specific USVs, and induced place preference for the treatment-paired compartment in the inflammatory pain model.

Finally, the side effect profile of mPD5 differed substantially from the current standard of care for chronic pain conditions, including both centrally and peripherally acting drugs. Taken together, these features advocate that mPD5 represents a compelling drug candidate for further preclinical testing before clinical trials and treatment of chronic pain.

## Methods

Further information can be found in Supplemental Methods.

### Sex as a biological variable.

Our study examined male and female mice, and similar findings are reported for both sexes.

### Study approval.

Experiments involving animals were performed in accordance with guidelines of the Danish Animal Experimentation Inspectorate (permission number 2016-15-0201-00976, 2021-15-0201-01036, 2020-15-020100439, and 2022-15-0201-01216) in a fully AAALAC-accredited facility under the supervision of local animal welfare committee. In all animal experiments, the experimenter was blinded to treatment, except for groups treated with 10 mg/kg morphine, since the “morphine tail” gives it away.

### Data availability.

Values for all data points in graphs are reported in the Supporting Data Values file.

## Author contributions

KLJ, CMG, GNH, and LS were involved in SNI experiments. KLJ performed CFA von Frey experiments and analysis on behavioral data. KLJ and LJF performed CFA anxiodepressive experiments. KLJ performed sePP and CPP experiments. KLJ performed USV experiments and EGP analyzed the data. CMG performed Barnes maze and open field experiments. CMG and KLJ performed capsaicin and tail immersion experiments. IBK, CH, CDT, ABIS, and MDC conducted the CIBP experiment and analyzed the data with KLJ. NRC and GNH performed and analyzed biochemical and biophysical experiments with assistance on SAXS beam time from FGT and model-based SAXS data analysis performed by LA. RCB and LS conducted experiments. MLT created the illustrations of PD5, mPD5, sPD5, and aPD5. SEJ performed whole-tissue clearing and immunocytochemical experiments followed by interpretation and visualization. GAH assisted with whole-tissue clearing experiments. AJ performed fertility experiments with help from FPM. KLM, ATS, and AMH supervised the research. KLJ, NRC, KLM, and ATS conceptualized the study, designed the research, and interpreted data. NRC and KLM invented the dimeric peptides. KLJ and KLM wrote the manuscript. All authors reviewed and critically evaluated the manuscript.

## Supplementary Material

Supplemental data

Supporting data values

## Figures and Tables

**Table 1:**
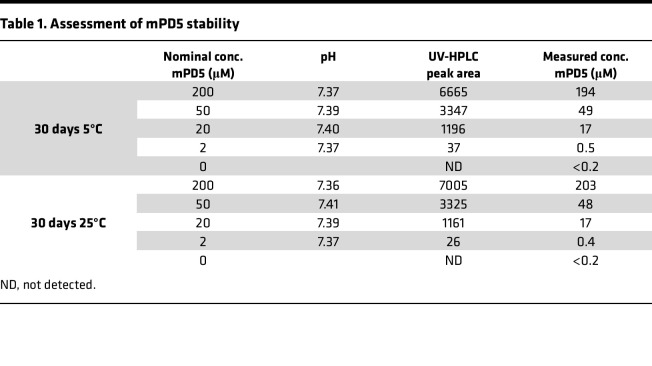
Assessment of mPD5 stability

**Table 2:**
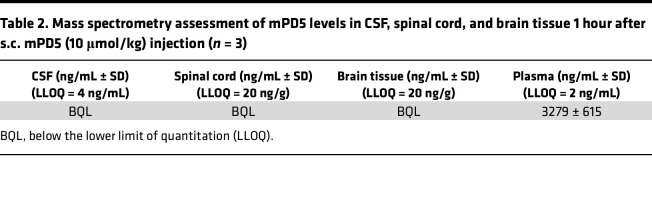
Mass spectrometry assessment of mPD5 levels in CSF, spinal cord, and brain tissue 1 hour after s.c. mPD5 (10 μmol/kg) injection (*n* = 3)

## Version 1 09/17/2024

In-Press Preview

## Version 2 10/22/2024

Electronic publication
